# Current Aspects on the Management of Perforated Acute Diverticulitis: A Narrative Review

**DOI:** 10.7759/cureus.28446

**Published:** 2022-08-26

**Authors:** Efstathios T Pavlidis, Theodoros E Pavlidis

**Affiliations:** 1 2nd Surgical Propedeutic Department, Hippocration Hospital, Aristotle University of Thessaloniki, School of Medicine, Thessaloniki, GRC

**Keywords:** septic shock, severe sepsis, perforation, acute surgical abdomen, emergency surgery, acute diverticulitis

## Abstract

Unhealthy nutritional habits and the current western lifestyle have led to an increased incidence of acute diverticulitis, which mainly affects older patients. However, the disease course in younger patients might be more severe. It has a continued increase in surgical practice, as it is the most common clinical condition encountered in the emergencies. Diagnosis and management have changed over the past decade. C-reactive protein > 170 mg/L represents the cut-off point between moderate and severe diverticulitis, and a CT scan is mandatory. It demands urgent surgical management and has high morbidity and mortality rate, especially in immunosuppressed patients, reaching up to 25%. According to the contemporary guidelines, there have been certain indications for conservative management and re-evaluation (administration of antibiotics, CT-guided drainage of the abscess, when it is > 4 cm). They include pericolic air bubbles or a small amount of fluid, absence of abscess within a distance of 5 cm from the affected bowel or abscess ≤4 cm. In other cases, Hartmann’s sigmoidectomy is the procedure of choice. An alternative choice, nowadays, is resection and primary anastomosis with or without diverting stoma, especially in younger patients. Laparoscopic lavage only versus primary resection has been performed in severe cases of Hinchey III or IV. Damage control surgery, possible open abdomen, and reoperation are recommended in severe sepsis. Hinchey’s classification may not be absolutely adequate, and several modifications have been proposed. Current classification criteria (CRP, qSOFA score) are more appropriate. The decision-making must be individualized depending on the hemodynamic status (septic shock), age, comorbidity, immune status, intraoperative findings, and MPI (Mannheim peritonitis index).

## Introduction and background

The diverticulosis of the colon increases with age, particularly after the fifth decade of life (50% above the age of 60 years old), whereas it is less common under the age of 40 years; however, the vast majority remains asymptomatic and only 20% of patients need some kind of treatment during the lifetime [1]. Given that two-thirds of octogenarians have diverticulosis and in combination with the continued growing of life expectancy, the cases of acute diverticulitis appear with increasing incidence. It is related to unhealthy nutritional habits and the current western lifestyle [1,2]. The disease affects mainly the sigmoid colon and older patients, but it can occur even in the younger patients with a more severe course [1,2]. Its ongoing growth in routine practice makes it the most often event of abdominal emergencies in older patients [2]. Not only many concepts and practices in the pathophysiology of sepsis but also the management strategy have changed over the past decade. Hinchey’s classification and its modifications express the severity of the disease [1-5].

Perforated diverticulitis is influenced by immunosuppression (chronic administration of corticosteroids, transplantation, neoplasms, chronic renal or liver failure, HIV/AIDS), which causes altered clinical course with mild symptoms, delaying the diagnosis, and demands urgent surgical management with high morbidity and mortality rates of 19%-25%, compared to 4%-6% in the general population [5]. In the USA, 2.7% of adults are immunocompromised, and the overall estimated annual cost of treatment for diverticular disease exceeds two billion dollars [5].

In acute diverticulitis, the rate of perforation reaches 10%. Several risk factors, such as high C-reactive protein (CRP), leukocytosis, diffuse abdominal pain and distension, inhibition of gas and fecal excretion, vomiting, steroid usage, a primary episode, and comorbidity, may predict underlying complications [3].

A multicenter study from Japan assessed the risk factors for perforated diverticulitis (fever above 38.5°C, the left colon affection, older age, and delay in the diagnosis). Another finding was that diverticulitis affected mainly younger patients between 40 to 60 years. The right colon location was more common (70%). The left colon location concerned mainly older patients (61%) [6].

Obesity, especially visceral obesity rather than high BMI, increases the likelihood of complicated diverticular disease [1,3]. A large national database analysis from the USA including a total of 2,019 patients with perforated acute diverticulitis showed that it did not affect the postoperative outcome after Hartmann’s operation. Morbid obesity (BMI > 35 kg/m2) was reported in 20.5% of the studied patients. Interestingly, high mortality or hospitalization rates were not observed in these patients compared with other non-obese patients, but only an increased reoperation rate was reported [7].

There are new guidelines by the EAES (European Association for Endoscopic Surgery) and the SAGES (Society of American Gastrointestinal and Endoscopic Surgeons) 2018 consensus conference, and the 2020 update of the WSES (World Society of Emergency Surgery) determining the proper management, i.e., conservative, interventional, or surgical [8,9].

A large nationwide study from Italy including 41,622 patients with complicated acute diverticulitis showed the highest hospitalization rate for patients ≥ 70 years of age. The admissions were higher for patients up to 60 years with a predominance of males, whereas females predominated in admissions for those over 60 years. There was an ongoing rate of hospitalization during the study period (2008 to 2015), particularly for younger patients; likewise, this increasing rate per year was assessed at 5.1% for peritonitis and 5.8% for abscess formation. The overall mean rate of peritonitis reached 35.5% [10].

This narrative review focuses on updating our knowledge on contemporary aspects in diagnosis, staging, and therapeutic options in diverticular disease, thereby highlighting the changes during the last 10 years.

## Review

Clinical indications and diagnostic tools

In perforated diverticulitis, except of the persistent pain that originates from the lower abdomen, other symptoms include systemic manifestations, i.e., high fever, tachycardia, tachypnea, malaise or arterial hypotension, and mental confusion in more severe cases. On clinical examination of the abdomen, there are signs of peritonitis (localized, tender mass, or diffused), including tenderness, muscle guarding, and rebound tenderness [1,2].

CT scan is the gold standard for establishing the diagnosis of acute diverticulitis and determining its severity [1,2]. Perforated diverticulitis is classified by the dimensions of abscess as well as the grade of the peritonitis either localized or generalized (purulent or fecal), as shown in Table 1 [2]. It can detect paracolic fluid collection, fistula with urinary bladder mainly or vagina, presence of intra-abdominal air, and obstructive ileus, and thus determine the indication for early surgical management. On admission, the radiological characteristics of a CT scan can predict the outcome of treatment. When the affected colon segment is >7 cm in length or there is an existence of an abscess > 1 cm in diameter, then the risk of conservative management failure will be higher [11].

**Table 1:** Classifications of diverticulitis and its CT findings [2]

Hinchey classification	Modified Hinchey classification	Accompanying CT findings
	Stage 0: clinically mild diverticulitis	Diverticula with or without wall thickening of the colon
	Stage Ia: confined pericolic inflammation and phlegmonous inflammation	Colonic wall thickening with inflammatory reaction in pericolic fatty tissue
Stage I: pericolic abscess or phlegmon	Stage Ib: abscess formation (<5 cm) in the proximity of the primary inflammatory process	Alterations as stage Ia + pericolic or mesocolic abscess formation
Stage II: pelvic, intra-abdominal, or retroperitoneal abscess	Stage II: intra‐abdominal abscess, pelvic or retroperitoneal abscess, abscess distant from the primary inflammatory process	Alteration as stage Ia + distant abscess formation (mostly pelvic or interloop abscesses)
Stage III: generalized purulent peritonitis	Stage III: generalized purulent peritonitis	Free air with local or generalized free fluid and possible thickening of the peritoneum (no open communication with bowel lumen)
Stage IV: generalized fecal peritonitis	Stage IV: fecal peritonitis	Free perforation, open communication with bowel lumen

In addition, it should be stressed that MDCT (multidetector computed tomography) is the tool of first selection for making accurate diagnosis and classification of diverticulitis including complications. Its diagnostic accuracy for the site of perforation reaches up to 85%. It can detect either direct findings (focal colon continuum wall lesions, presence of pericolic gas, and leak of contrast material) or indirect findings (segmental colon wall thickening, abnormal wall enhancement, pericolic fat edema, fluid collection, or abscess). Additionally, MDCT can differentiate, with high rates of accuracy, between complicated colon diverticulitis and colon cancer, which is a possible co-existence [12]. Ultrasound (US) and contrast-enhanced US exhibit similar results to CT and can be helpful, especially in pregnancy, as well as MRI (magnetic resonance imaging) [12].

Inflammatory markers

On admission, CRP is the most useful inflammatory marker in daily practice for the severity prediction of acute diverticulitis. CRP ≥150 mg/L is an incriminating factor with strong possibility for perforated diverticulitis, especially in advanced age, and a CT scan is required necessarily. In addition, it has a high risk of death in combination with free abdominal fluid in CT [13]. CRP > 50 mg/L indicates uncomplicated acute diverticulitis, but CRP > 170 mg/L represents the cut-off point between moderate and severe diverticulitis. white blood cell count and erythrocyte sedimentation rate also have a significant role. Other biomarkers such as fecal calprotectin, serum bilirubin, ﬁbrinogen, alkaline phosphatase, α-1 acid glycoprotein, and matrix metalloproteinase and their inhibitors contribute little toward complicated acute diverticulitis [14].

Staging systems

Hinchey’s staging system, which has been the most commonly used system in the past three decades, is not absolutely adequate according to the recent data. Current classification criteria (CRP, quick Sepsis-related Organ Failure Assessment [qSOFA] score) are more appropriate. Hinchey’s classification and its modification with the accompanying (CT) findings is shown in Table 1 as referred by Tochigi et al. and Kaiser et al. [2].

The 3rd International Consensus Definitions for Sepsis and Septic Shock (Sepsis-3) determined the following update [15].

Sepsis means a life-threatening multiple organic malfunction due to SIRS (systemic inflammatory response syndrome) resulting from infection.

SIRS is detected when at least two of the following are present: fever over 38°C or under 36°C, tachycardia over 90/min, tachypnea over 20/min or pCO2 under 32 mm Hg, white blood cells over 12,000 /mm or under 4,000/mm, or their immature forms over 10%.

Organic malfunction can be indicated by SOFA (Sepsis-related Organ Failure Assessment) score ≥ 2 points, with mortality exceeding 10%.

The on-time detection of possible sepsis, without any delay in its management, is based on a qSOFA score when at least two out of the following three clinical indications are present: tachypnea ≥ 22/min, low level of consciousness, and arterial pressure ≤ 100 mm Hg, as shown in Table 2.

**Table 2:** Quick SOFA score [15]

Parameter	Value
Respiratory rate	≥22/min
Consciousness level	low
Arterial pressure	≤100 mm Hg

Septic shock means aggravation of sepsis, which is characterized by the need for vasoconstrictors to maintain a mean blood pressure ≥ 65 mm Hg and blood lactates above 2 mmol/L or 18 mg/dL given that there is an absence of hypovolemia. It is associated with mortality > 40%.

MPI (Mannheim peritonitis index) is a scoring tool of severity used for the staging of peritonitis and predicting mortality, as shown in Table 3 and Table 4, as suggested by Muralidhar et al. [16].

**Table 3:** Mannheim peritonitis index [16] *Definitions of organ failure - kidney: creatinine > 177 μmol/L, urea > 167 μmol/L, oliguria < 20 mL/h; lung: pO2 < 50 mm Hg, pCO2 > 50 mm Hg; shock: hypodynamic or hyperdynamic; intestinal obstruction, only if profound; paralysis > 24 h or complete mechanical ileus

Risk factor	Weightage, if any score
Age >50 years	5
Female gender	5
Organ failure*	7
Malignancy	4
Preoperative duration of peritonitis >24 hours	4
Origin of sepsis not colonic	4
Diffuse generalized peritonitis	6
Exudates	
Clear	0
Cloudy, purulent	6
Fecal	12

**Table 4:** Assessment of severity of peritonitis using the Mannheim peritonitis index [16]

Score	Mortality rate	Morbidity rate
<21	0%	13.33%
21-27	27.28%	65.71%
>27	100%	100%

Conservative and interventional treatment

According to the recent recommendations by the EAES, SAGES [8], and WSES [9], conservative treatment, as for uncomplicated acute diverticulitis (antibiotics and re-evaluation, both clinical and diagnostic), is indicated for stage Ia (air bubbles around the colon or restricted fluid in small amount in a distance of 5 cm from the affected colon, but no presence of abscess) and stage Ib (abscess around the colon equal or less than 4 cm in diameter and in a distance of 5 cm from the affected colon). The conservative treatment plus CT-guided drainage is indicated for stage IIa (abscess presence more than 4-5 cm in diameter and distant abscess more than 5 cm from the primary focus). For stage IIa, an early colonic evaluation is suggested within 4-6 weeks.

It is crucial on admission the urgent administration of wide-spectrum antibiotics without any delay, along with appropriate hydration, electrolyte balance, and inotropic drugs if needed. A septic patient requires fast preoperative relevant stabilization and evaluation as well as intensive care unit (ICU) postoperatively. The failure of any improvement after the first recovery efforts constitutes a strong indication for emergency surgery despite other diagnostic findings (imaging, biomarkers) [2].

The recommended antibiotics can be used either as single agent or combination of agents. The treatment by a single agent includes ticarcillin-clavulanic acid, moxifloxacin, ertapenem, tigecycline, and cefoxitin for mild or moderate severity cases, and meropenem, imipenem-cilastatin, piperacillin-tazobactam, doripenem for high-risk patients or those with severe inflammation. A combination regimen with metronidazole could be administered for mild or moderate severity cases (cefuroxime, cefazolin, cefotaxime, ceftriaxone, levofloxacin, ciprofloxacin), and ceftazidime, cefepime, levofloxacin, and ciprofloxacin for high-risk patients or those with severe inflammation) [17].

Conservative treatment of stage Ia acute diverticulitis with sequestered air around the colon is safe, feasible, and effective in 95% of such cases [18], as well as in cases with a distant intra-abdominal air in small amount or local air around the colon but without signs of peritonitis [19]. In the case of pericolic air without clinical signs, successful antibiotic treatment reaches up to 94% [20]. However, large amounts of distant free air indicate rather diffuse peritonitis, and must be managed surgically, whereas isolated pericolic air is amenable to non-operative management. Further well-designed randomized controlled trials might be useful in clarifying this issue.

When distant free gas is present on CT scan but with no evidence of generalized intra-peritoneal fluid collection, a conservative management, after selection of cases, must be considered only if the option of close follow-up is available. Up to 25% of cases that are initially managed conservatively may require urgent operative intervention [9].

Pelvic abscesses are less accessible than pericolic ones, making CT-guided drainage riskier. Thus, laparoscopic procedure with deep draining is a safe and effective minimal invasion choice [21].

Immunocompromised patients have a greater probability of failure with the usual conservative therapy and require urgent surgical intervention despite the high mortality [9].

Operative therapy

Operative management is indicated for stage IIb (remote gas in a distance more than 5 cm from the primary focus, stage III (diffuse purulent peritonitis), stage IV (evidence of fecal peritonitis), failure of conservative treatment, and gradual clinical deterioration [8,9]. Hartmann’s sigmoidectomy is the procedure of choice, but there are several other choices nowadays.

The choice of surgical procedure depends on the intraoperative findings, hemodynamic status, presence of sepsis or severe septic shock with organ dysfunction, physical status, age, comorbidities, and the immune status; qSOFA score ≥ 2 and Mannheim peritonitis index (MPI) > 20 assist in determination of staging in high-risk patients. Urgent laparoscopic procedure might have advantages compared to open surgery in hemodynamically stable patients, which includes sigmoidectomy with anastomosis, laparoscopic lavage, and Hartmann's procedure. In septic patients and severe peritonitis, open surgery is required: either Hartmann’s procedure as first-line treatment or damage control surgery (DCS) in case of hemodynamic instability [17,22].

Although the standard choice for fecal peritonitis is Hartmann's procedure, the best choice for purulent peritonitis is still uncertain, and randomized trials are in progress [23,24].

Laparoscopic lavage and drainage is indicated only in meticulously selected patients with diffuse peritonitis. In cases with severe sepsis and peritonitis, this approach is not recommended as the treatment of choice [9].

A recent systematic review including a meta-analysis of randomized controlled trials found that laparoscopic sigmoid resection does not prevail compared to the open procedure, taking into account the postoperative morbidity and mortality rate. Also, laparoscopic lavage is as safe as a resection [25].

Postoperative antibiotic therapy in case of adequate source control is recommended for a four-day period [9].

The indications for emergency surgical management in the elderly are difficult to be defined. It all depends on organ function status and the capacity to overcome surgical stress. A study from Japan on octogenarians undergoing urgent open operation following perforated acute diverticulitis showed that the mortality was 100% in cases having MPI ≥ 26 and 46% in cases having an ASA score of ≥ 3. These cases require great caution in making choice of surgical procedure and the best supportive care in the ICU [26].

Choices of surgical treatment

Primary Anastomosis Accompanied by Stool Diversion or Not After Sigmoidectomy Instead of Hartmann’s Operation

Nowadays, the choice of traditional Hartmann's operation is debatable, but the choice between primary anastomosis or not after sigmoidectomy still remains controversial in daily practice [27]. The probability of stoma closure restoring the bowel continuity after the primary anastomosis is higher, easy to perform, and accompanied by lower morbidity and mortality, which has caused this debate.

However, in any case, there is a strong recommendation for Hartmann’s operation in the treatment of patients having generalized peritonitis, critical illness, and multiple comorbidities [9].

The LADIES trial, a multicenter, randomized one, assessed Hartmann's operation instead of sigmoidectomy accompanied by primary anastomosis and diverting ileostomy or not for stage III and IV generalized peritonitis, which included 130 patients. It showed that the one-year survival without ileostomy was better for cases undergoing anastomosis instead of those undergoing Hartmann's operation, although no significant findings were assessed between them in rates of postoperative morbidity and mortality. Thus, the recommendation is for primary anastomosis instead of Hartmann's operation in hemodynamic stable immunocompetent patients aged < 85 years old suffering from purulent and fecal peritonitis [28].

A recent systematic review and meta-analysis including 918 patients confirmed the results of the previous study in favor of primary anastomosis instead of Hartmann's operation. Primary anastomosis in hemodynamic stability for stage III or even IV peritonitis ensures reduced both stoma remaining for a long time and morbidity compared to Hartmann's procedure [29]. Likewise, another recent systematic review agrees with that and indicates clearly that primary anastomosis should be the preferred choice instead of Hartmann's procedure in selected stable cases of purulent or fecal peritonitis [30].

A multicenter randomized controlled trial conducted by DIVERTI from France included 102 patients and provided a piece of additional evidence in favor of anastomosis accompanied by defunctioning ileostomy for stage III or even IV peritonitis over Hartmann's operation. The rate of stoma closure was found to be greater for anastomosis, whereas the mortality was similar in both the groups [31]. This trial has been updated recently by assessing the long-term (median time of more than nine years) outcomes. Primary anastomosis led to lesser complications, better quality of life, fewer incisional hernias, and reoperations than Hartmann’s procedure [32].

A multicenter randomized clinical trial from Switzerland assessing anastomosis with diverting ileostomy or Hartmann's operation in case of generalized peritonitis including 62 patients showed better results in favor of the primary anastomosis [33].

Before the guidelines of the American Society of Colon and Rectal Surgeons in 2014, a nationwide trial from 1998 to 2011 included 124,198 patients with sigmoidectomy for acute diverticulitis. Only 3.9% underwent anastomosis with a diverting ileostomy. The vast majority underwent end colostomy. The complication rate was higher (32.1% vs. 23.3%) as well as the in-hospital mortality (16.0% vs. 6.4%) for primary anastomosis with diversion compared with end colostomy. These disappointing results following anastomosis accompanied by stoma may have discouraged the surgeons to follow the new guidelines despite a relevant increase after 2014 [34].

Another nationwide analysis from the USA including 2,729 patients showed that most surgeons preferred Hartmann's procedure and only 7.6% performed anastomosis accompanied by ileostomy, which was considered a reasonable option but in stable patients [35].

The choice of loop ileostomy or colostomy for diverting stoma is a conflicting aspect but is left to the surgeon’s discretion.

Timing for restoration of bowel continuity following Hartmann's operation is controversial and complex and is associated with increased encountered complication rates. A retrospective multicenter trial from Israel showed that the median time for restoration was six months [36].

Laparoscopic Sigmoidectomy

The suggestion of emergency performance of this procedure in cases with stage III or IV due to perforation is applicable only if there is availability of equipment and adequate operative training [9]. Thus, it is indicated in cases after selection [4].

The recent major advances challenged the traditional approach of Hartmann's operation by performing laparoscopic anastomosis; this approach offers clear advantages and excellent results for feculent peritonitis (Hinchey IV) provided that an accurate patient selection, hemodynamic stability, and postoperative enhanced recovery protocol are achieved [37].

The laparoscopic choice has better results compared to the open technique in both elective cases for an uncomplicated diverticulitis and emergency cases for complicated diverticulitis. In the emergency, complicated diverticulitis case, experienced surgeons must perform all procedures, i.e., Hartmann's procedure, sigmoidectomy accompanied by primary anastomosis, and reversal of end colostomy after Hartmann's operation [38].

Laparoscopic sigmoid resection excels the open sigmoid resection for complicated acute diverticulitis in terms of morbidity and hospitalization [39,40]. Laparoscopic treatment is recommended for the management of many other intraperitoneal inflammations [41].

Damage Control Surgery

This innovation was introduced initially for the management of severe trauma, but over the last decade, it has also been performed as a life-saving treatment in critical illness septic cases with intra-abdominal origin. DCS and reoperation is reserved for cases without hemodynamic stability because of severe generalized peritoneal infection. The recent recommendation for this choice is only in selected cases with critical illness who could not cope with great surgical intervention [9].

In septic cases with severe intraperitoneal inflammation, the mortality exceeds 80%. Although the fascial closure has been preferred as the current standard, many others choose the rapid controlling of the septic situation and leaving the abdominal cavity open to improve the outcome [42]. Rapid antibiotics and urgent operation for controlling the sepsis are important for effective management. A prospective randomized controlled trial “The Closed Or Open after Laparotomy (COOL) study” found that an open abdomen and VAC (vacuum-assisted closure) option in order to eliminate the reactive exudates and improve the SIRS manifestations is a valuable choice [42]. However, a recently published study from the USA including 460 patients who were operated on for Hinchey IV diverticulitis showed opposite results. However, urgent operation for sepsis control or open abdomen instead of fascial stitch showed longer hospital stay and greater need postoperatively for special care to rehabilitate indicating its restriction routinely but reserving this to selective use only [43].

An analysis of eight retrospective trials on DCS of 256 cases confirmed DCS as an effective option for stage III and IV perforation, allowing a great rate of anastomosis and thus avoiding colostomy in above 50%. It is performed in two stages. First, urgent surgical operation focuses on restricted sigmoidectomy including the affected segment, closure of both stumps, enough peritoneal washing, and VAC use. After elapse of 24 to 48 hours and appropriate resuscitation follows the second surgery either for definite reconstruction of colorectal continuity (accompanied by defunctioning ileostomy or not) or Hartmann's operation [44].

Likewise, another review including 359 patients with most, i.e., 81.6% having ASA score III or IV, showed that DCS had a good outcome in terms of overall morbidity (23%-74%), 30-day mortality (0%-20%), and overall mortality at follow-up (7%-33%), as well as a lower rate of definitive stoma than Hartmann's procedure [45].

Another trial confirmed the aforementioned, but the heterogeneity of included patients makes mandatory the construction of more randomized controlled trials [46]. Also, similar results for DCS are obtained by other studies [47-49].

The presence of ongoing peritonitis in the second operation after DCS predicts worse outcome [50].

Laparoscopic Lavage

Laparoscopic intra-abdominal washing accompanied by drains is performed since it is a safe and effective option compared to colonic resection because it rather offers stoma avoidance in patients with diffuse peritonitis. Furthermore, this technique ensures aspiration, abdominal lavage, and drain placements [9,25]. Promising results provide a laparoscopic lavage in combination to direct suturing of perforation and drains [51].

A recently published international clinical trial from 2010 to 2014 included 199 patients with purulent peritonitis (exclusion of fecal peritonitis). The patients received either laparoscopic peritoneal lavage or colectomy and had a long-term follow-up between 2018 and 2019 with encouraging results. The short-term outcome showed no difference in either lavage or resection group, severe complications (35% vs. 36%), and overall mortality, 32% vs. 25%, respectively. Long-term follow-up revealed no difference in severe consequences. After laparoscopic lavage, recurrent diverticulitis prevailed (21% vs. 4%), respectively, often leading to sigmoid resection [52]. Further analysis of the results from the previous study showed that laparoscopic lavage was accompanied by deeper infections of the surgical site and subsequent interventions in case of persistent sepsis [53]. Furthermore, it does not restrict severe consequences after surgery thus leading to unacceptable results [54]. The above outcome argues against the use of laparoscopic lavage.

On the other hand, another trial including 66 cases of purulent peritonitis or a pelvic abscess not accessible for imaging-guided intervention, in which either laparoscopic lavage or laparoscopic sigmoidectomy was performed, found conflicting outcomes. Therefore, laparoscopic lavage is often questioned concerning reoperation, postoperative ongoing sepsis, and recurrence [55]. The procedure should be standardized by expert surgeons, and the results from randomized clinical trials, which have been in progress, must confirm its use [56].

Another trial coming from 24 centers between 2005 and 2015 included 404 consecutive patients with purulent peritonitis because of perforated acute diverticulitis and managed by laparoscopic lavage. A high rate of controlling sepsis successfully (74.5%) in cases after selection was observed, accompanied by low operative mortality (1.9%), reoperation (13.7%), and recurrence (26.7%). Factors for recurrence were previous acute diverticulitis, female gender, and younger age. The selection criteria for laparoscopic lavage included patients with lower MPI score (<24) and ASA score (<3), no evidence of uncovered perforation, not enough manipulations for adhesiolysis, and no history of acute diverticulitis. [57].

Likewise, randomized clinical trials comparing laparoscopic lavage versus Hartmann's operation for purulent peritonitis showed that laparoscopic lavage is a better, safe, and effective alternative than Hartmann’s procedure [58,59]. Also, a meta-analysis confirmed the validity of laparoscopic lavage [60].

The urgent laparoscopic lavage is proposed as a "bridge" of the elective laparoscopic sigmoidectomy later, thus avoiding Hartmann’s operation in the emergency situation. This approach must be reserved for patients without systemic toxicity and should be performed by experienced surgeons only [61].

A review and meta-analysis including 589 patients compared laparoscopic lavage and resection. It showed that the presence of an affected colon in laparoscopic lavage had the same mortality, but a three-fold higher risk for persistent peritonitis, intraperitoneal abscesses, and urgent surgery [62], although two other meta-analysis studies confirmed higher abscess formation requiring drainage intervention, no difference in mortality, and serious morbidity [63,64].

Right-sided diverticulitis

While diverticulitis located in the left side is common in Western countries, the right-sided location is rare. A much recent study from Israel found that among 1,000 admissions with diverticulitis, only 10% had right-sided location affecting mainly the cecum. The clinical picture was characterized mainly by right lower quadrant abdominal pain and benign course without major complications, with low mild complication (10%), and with low recurrence rates (3%). The conservative treatment was adequate without the need for surgery [65].

Left-sided perforated diverticulitis is more severe, especially in the elderly, than that of the right side [66]. Although the complications that require surgical intervention are more in cases with a left location compared to cases with a right location, the diagnosis and management policy is identical.

All of the aforementioned observations and recommendations concerning location of diverticulitis in the left side are also valid for the right side [9]. The prediction of sepsis is positive when two or more signs are present.

## Conclusions

Within the limitations of a narrative review, we could summarize that CT scan with contrast is the first choice imaging to establish accurate diagnosis of the disease and its staging in acute setting. CRP is the most useful inflammatory marker in daily practice for the severity of acute diverticulitis as well as the qSOFA score. Conservative treatment is indicated for modified Hinchey stage Ia and Ib and plus interventional drainage for stage IIa. Operative management is indicated for stage IIb, stage III (generalized purulent peritonitis), and stage IV (fecal peritonitis). Hartmann’s sigmoidectomy was the procedure of choice, but it is still recommended strongly for generalized peritonitis in critical cases and for those suffering from various co-existing diseases. Primary anastomosis accompanied by diverting stoma or not, laparoscopic sigmoidectomy, DCS, and laparoscopic peritoneal lavage with drainage are the other updated options. Primary anastomosis in hemodynamically stable patients avoids persistent end colostomy and morbidity. DCS saves lives in critically ill patients and reduces stoma. Laparoscopic lavage is indicated only for those without systemic toxicity and must be performed by experienced hands, but there is a debate not supporting its use. The decision of the best choice is controversial, must be individualized, and depends on hemodynamic status, septic shock, qSOFA score, age, comorbidity, immune status, intraoperative findings, and MPI. Ongoing randomized controlled trials possibly may clarify this complicated issue.
